# Childhood to Adult Neurodevelopment in Gene-Expanded Huntington’s Disease (ChANGE-HD): A prospective longitudinal neurodevelopmental study of Huntington’s disease

**DOI:** 10.1371/journal.pone.0336088

**Published:** 2026-06-25

**Authors:** Mohit Neema, Nabil Halabi, Michael V. Freedberg, Peggy C. Nopoulos, the ChANGE-HD investigators, coordinators, and consultants

**Affiliations:** 1 Department of Psychiatry, Carver College of Medicine at the University of Iowa, Iowa City, Iowa, United States of America; 2 Department of Neurology, Carver College of Medicine at the University of Iowa, Iowa City, Iowa, United States of America; 3 Stead Family Department of Pediatrics at the University of Iowa, Iowa City, Iowa, United States of America; University of Tartu, ESTONIA

## Abstract

Although adult Huntington’s disease (HD) studies have significantly advanced our understanding of the course of degeneration, they may underrepresent critical neurodevelopmental aspects of the disease. Significant gaps remain in understanding how mutant huntingtin affects early neurodevelopment, its long-term impact, as well as potential implications for treatment outcomes. The Childhood to Adult Neurodevelopment in Gene-Expanded Huntington’s Disease (*ChANGE-HD;* NCT01951588) study aims to evaluate brain structure and function in premanifest, at-risk children and young adults, and explore HD’s developmental origins. Here, we introduce the ChANGE-HD study, which will investigate and integrate the neurodevelopmental and neurodegenerative aspects of HD. The ChANGE-HD study is a prospective, seven-year multi-site observational study with an accelerated longitudinal design, where participants are not bound to a fixed schedule across multiple visits. Four hundred and fifty participants aged 6–30 years who are at risk for HD will be recruited and asked to return for multiple visits (if possible). At each visit, cognitive, motor, behavioral, blood/saliva, and MRI data are collected. Alongside ChANGE-HD, we are also recruiting individuals for the juvenile-onset HD (JOHD) study to investigate the neuropathology of this rarer form of HD. ChANGE-HD represents the first prospective multi-site study to systematically document brain structure and function during the premanifest phase of HD in children and young adults. Data collection is ongoing with first results anticipated in 2026–2027. The ChANGE-HD approach is likely to provide novel physiological insights and guide the development of therapeutic strategies tailored to both the developmental and degenerative phases of the disease.

## Introduction

Huntington’s disease (HD) is an autosomal-dominant neurodegenerative disorder of the central nervous system (CNS) resulting from an abnormal trinucleotide repeat expansion of cytosine-adenine-guanine (CAG) in the Huntingtin (*HTT*) gene [1]. In the general population, the number of CAG repeats encoded within exon 1 of *HTT* ranges from 10 to 35. Repeat lengths between 36 and 39 demonstrate variable penetrance, whereas repeats exceeding 39 are classified as mutant *HTT (mHTT)*, resulting in HD with full penetrance.

*HTT* is a highly conserved gene essential for nervous system development, neuronal stability, and survival. The gene is maintained across species [2], suggesting that CAG repeats within *HTT* have undergone positive selection, potentially driving species survival through increasing CNS complexity. Notably, humans exhibit the highest number of CAG repeats, underscoring their unique evolutionary significance to the development of higher-order cognitive tasks [3–5].

Traditionally, HD has been viewed as a neurodegenerative disorder primarily affecting the striatum, characterized by motor, cognitive, and behavioral dysfunctions [6]. The prevailing explanatory hypothesis attributes the disease to a toxic gain-of-function mutation within *mHTT* (i.e., the CAG repeat expansion), resulting in neuronal damage and cell death. However, emerging stem cell, animal, and human neuropathological evidence suggests that brain development changes are critical to HD’s pathoetiology [7–9], raising the hypothesis that HD is an unintended outcome of an adaptive evolutionary process supported by a more functional CNS [3]. Thus, *mHTT*, in addition to causing pathological effects in adulthood, may positively affect the course of neurodevelopment. Understanding the complex interplay between neurodevelopment and neurodegeneration could transform treatment approaches, particularly by identifying critical inflection points where neurodevelopment transitions into degeneration. Most studies show that the degenerative process in HD begins roughly 20–25 years prior to onset [10–12]. These findings underscore the need to understand the effects of *HTT* on development, particularly in children and young adults at risk for HD.

### Lessons from Kids-HD

In 2009, the National Institute of Health (NIH) -funded Kids-HD study (ClinicalTrials.gov Identifier: NCT01860339) was launched at the University of Iowa, investigating the impact of *mHTT* on brain development in children at risk for HD [7]. For research purposes only, children aged 6–18 years-old at risk for HD (defined as having a parent/grandparent with HD) were genotyped and categorized as gene-expanded (i.e., GE, CAG >= 36) or gene-non-expanded (i.e., GNE, CAG < 36). The GNE children served as an excellent control group, given that being raised in an HD family can produce unique psychosocial strain not captured by children of non-HD families. A separate group of age-matched participants with no familial link to HD was also recruited as a comparison group. The Kids-HD study modeled the progression of HD several decades before the predicted age of motor onset in the GE participants. In total, we collected data from 406 individuals (13.73 ± 4.33 year; 177 Males, 227 Females; 2 unspecified) across 663 visits. Over the years, the Kids-HD study produced several key findings (Table 1). Some highlights include:

**Table 1:** Summary of Kids-HD papers/findings.

Clinical outcomes		
**Reference**	**Objective**	**Results/Conclusions**
Lee et al., 2018 [20]	Assess CAG effect on cognition in GE and GNE children aged 6–18 years.	*mHTT* has an inverted U-shaped effect on general intelligence (GAI): GAI scores increased and decreased with repeat length before and after 40–41 repeats, respectively, suggesting antagonistic effect of *HTT* on intelligence.
Tereshchenko et al., 2020 [21]	To determine the impact of *mHTT* on human measures of growth, including height, weight, and body mass index (BMI), between child and adolescent carriers of *mHTT* and control peers.	Decades before HD symptom onset, physical growth is abnormal in adolescent carriers of *mHTT*, suggesting that developmental aberrations may be systemic and a vital part of disease pathology.
Schultz et al., 2021 [14]	Evaluate the trajectory of cognitive skill development as a function of CAG repeat length in children and adolescents that carry the mutation that causes HD.	The development of verbal skills plateaus earlier as CAG repeat length increases.
Reasoner et al., 2022 [22]	Compare neuropsychiatric symptoms between child and adolescent huntingtin gene-mutation carriers and noncarriers	GE children had lower depression/anxiety compared to their peers. In contrast, GNE individuals largely show striatal volume growth. These findings suggest that lower depression and anxiety scores in GE children and adolescents may be associated with differential striatal growth.
Schultz et al., 2021 [23]	Compare crude markers of autonomic nervous system (ANS) function between GE children who were decades from their predicted motor onset and GNE children.	GE individuals 15–25 years before motor symptom onset have higher resting heart rate, systolic blood pressure, and core body temperature than their peers and GE individuals less than 15 years before onset. ANS dysfunction in HD seems to occur approximately 20 years prior to the predicted onset of motor symptoms of HD.
Byrne et al., 2017 [24]	Investigated whether neurofilament light protein (also known as NfL) in blood is a potential prognostic marker of neurodegeneration in patients with HD.	*mHTT* carriers have higher mean concentrations than non-carriers that increases across disease stages. NfL plasma concentrations were associated with clinical and MRI findings.
Neema et al., 2024 [17]	Investigate antagonistic pleiotropy in HD using a years-to-onset paradigm in a unique sample of children and young adults at risk for HD.	Decades from motor onset, GE participants show larger cerebral volumes and cortical features and significantly better cognitive, behavioral, and motor scores versus GNE controls. Greater brain size was associated with better behavioral performance in GE individuals.
**Neuroimaging outcomes**		
**Reference**	**Objective**	**Results/Conclusions**
Lee et al., 2017 [13]	Evaluate CAG effect on brain structure and function in healthy controls aged 6–18 years.	CAG repeats are positively and negatively associated with brain structure changes and higher IQ in females and males, respectively.
van der Plas et al., 2019 [15]	Evaluate brain growth trajectories in child and adolescent carriers of *mHTT* compared to peers without the mutation.	GE individuals have greater striatal and pallidal volume compared with GNE, suggesting atypical brain development. CAG repeat length is positively correlated with early life striatal volume and a steeper slope of decline later in life.
Tereshchenko et al., 2020 [25]	Compare functional connectivity between the striatum and cerebellum in GE and GNE children at risk for HD.	Striatal-cerebellar resting state functional connectivity is greater in GE compared to GNE between in individuals 6–12 years old and continues to decline linearly afterwards.
Reasoner et al., 2022 [16]	Evaluate the impact of *mHTT* on the developmental trajectories of cortical thickness and surface area.	Cortical, but not striatal, volume is similar between GE and GNE individuals across age and not affected by CAG repeat length.

- ***HTT* impacts brain structure and function along the entire spectrum of repeats**: Higher repeats are associated with better cognitive skill and greater brain volume in GE and GNE individuals [13,14].
- **Methodological challenges of modeling age and CAG separately, versus years-to-onset model (which incorporates both):** The Kids-HD study employed an accelerated longitudinal design (ALD), the gold standard for evaluating brain development in children, which combines cross-sectional and longitudinal data to model age-related changes across a broad developmental window. This model showed not only interesting findings of accelerated striatal development, where GE children reached peak striatal volume years before the GNE group, but that these trajectories were profoundly affected by CAG repeat length [15]. We also found that every CAG repeat conferred a greater advantage in general cognitive ability [14]. This effect peaked at repeats of 43 and individuals with longer CAG repeats showed trajectories that were declining in function after age 15, likely representing changes due to the degenerative phase of the disease. The age-trajectory modeling results highlighted the profound consequences *HTT* and CAG repeat-length has on neurodevelopment. These findings recapitulate the well-established negative relationship between CAG repeat length and age of motor onset and require an updated model to take both factors, and their interactions, into account. Given the influence CAG repeats and age exert on longitudinal brain development, we began modeling the data in a Years-To-Onset (YTO) fashion to evaluate the time course of the disease from development to degeneration. The YTO model predicts the age of motor onset based on the participant’s current age and CAG repeat length. Importantly, the YTO model can reveal information about disease trajectory that is obscured when plotting data by age. For example, Reasoner et al. (2022) found that cortical volumes are similar across age groups [16], while Neema et al. (2024) found evidence for higher and lower cortical volumes depending on the YTO of the individual [17]. The critical difference between approaches is that while higher age and lower YTO both predict decline in cognitive and motoric function, age-only models ignore variability in disease trajectory caused by CAG repeat length. An example of the difference between an age-only trajectory and a YTO model is as follows: An 8-year-old with a CAG repeat length of 48 and a 22-year-old with a CAG length of 43 both have 26 YTO, despite being on opposite ends of the age trajectory model. Thus, potential differences in cortical volumes could have been missed by Reasoner et al. (2022) because participants at similar ages were at different points in their disease trajectory. Additionally, when evaluating a biomarker of degeneration in Kids-HD (neurofilament light; “NfL”), we found that NfL levels are normal as far back as 40 YTO but rise around 20 YTO [18]. These findings support the notion that the YTO model can accurately characterize the developmental phase of the disease process, which occurs 20 years before the onset of motor symptoms.
- **The antagonistic pleiotropy theory of *HTT* and its implications for neurodevelopment:** In our most recent publication, we revealed that GE individuals from 50−20 YTO have larger cerebral volumes, more cortical surface area, and higher IQs compared to GNE subjects, but the opposite is true after 20 YTO [17]. Striatal and pallidal volumes remain comparable in volume to GNE until 20 YTO after which they dramatically decline in volume, consistent with early vulnerability of the basal nuclei. These findings challenge the traditional concept of HD as a uniquely neurodegenerative disorder and highlight the neurodevelopmental role of *mHTT.* Additionally, Kids-HD research uncovered potential early advantages of *mHTT* in GE children, such as larger brain development that supports enhanced cognitive development and reduced depression/anxiety. However, these children will indeed develop HD, highlighting the yoking of advantage with disadvantage. Our findings support the notion of antagonistic pleiotropy in HD, where the same neurodevelopmental differences that confer early advantage eventually lead to vulnerability and subsequent degeneration [19]. Collectively, they reshape our understanding of the evolutionary implications of *mHTT* and its potential role in brain development

### Beyond Kids-HD: Embracing a new era with ChANGE-HD

While the Kids-HD study provided novel insights, it also raised several critical questions that required further investigation. Kids-HD’s modest single-site sample (202 GE children) produced valuable data but required a necessary expansion for greater reliability and broader application. This prompted the launch of the NIH-funded, multi-site ChANGE-HD study in 2019, which builds upon the Kids-HD findings and addresses the following key areas:

- **Expansion and Replication:** No single study can provide definitive conclusions. ChANGE-HD aims to reproduce the Kids-HD findings using a larger sample size across multiple sites to ensure more robust, generalizable data.
- **CAG-specific effects:** Kids-HD identified CAG-specific effects in the GE group, where different repeat lengths produce distinct neurodevelopmental trajectories. To fully understand these effects, ChANGE-HD will recruit a larger cohort to enable a thorough analysis across a fuller range of repeats.
- **Wider age range amongst participants:** Kids-HD originally recruited participants from age 6 through age 18, but maturational changes extend into late 20s and early 30s [26]. Although YTO models address the strict need for sampling participants across a broad spectrum (as demonstrated by the example above), it is still beneficial to examine older participants who are approaching their date of motor onset to better model trajectories at low YTOs.
- **Evaluation of serum biomarkers of disease progression – NfL:** Serum NfL is a marker of neuronal damage in premanifest HD (among other neuronal diseases and disorders), with levels rising approximately 15–20 YTO, and is strongly correlated with the age of motor onset. Although NfL is classically absent in the developmental phase and appears after the onset of subtle neurodegeneration, its use as a biomarker for HD remains unclear and requires further investigation. The CHANGE-HD study aims to clarify the trajectory of NfL in younger at-risk individuals and explore its relationship with clinical outcomes and MRI findings.
- **The ChANGE-Juvenile Onset Huntington’s disease (JOHD) Study:** Expanding our sample size across multiple investigation sites allows us to delve deeper into the 5–10% of HD cases where symptom onset occurs before the age of 21, or JOHD [27–29]. Therefore, along with studying adolescent and adult-onset HD, we have included a companion study of ChANGE-JOHD where we will investigate the neuropathology of this rarer and more aggressive form of HD. This study will leverage the infrastructure of the ChANGE-HD study and follow a similar protocol.

The study also seeks to expand on other HD-related studies, such as TRACK-, PREDICT-, IMAGE-HD, and HD-YAS. Beyond the fact that we are studying a population that is much further from the possibility of disease manifestation, ChANGE-HD has other distinctions. First, whereas TRACK-, PREDICT-, and IMAGE-HD examined the disease from the earliest point of neurodegeneration and just prior to cortical and subcortical volumetric atrophy, the ChANGE-HD study focuses on developmental alterations in neural connectivity and structure, with the hypothesis that *mHTT* confers a pre-manifest benefit to inheritors. Second, whereas other studies have sought to determine the earliest potential time for interventions we seek to determine whether there is a point where early intervention may be detrimental. Third, we expand on HD-YAS by determining whether increases or decreases in cellular stress markers, such as NfL can be observed during the neurodevelopmental phase of the disease. Fourth, our broad toolbox of instruments has been updated to include more sensitive measures that can capture performance variability in individuals < 10 years old, such as the Q-Motor test, which relies on force transducers, rather than observation, to quantify motor system function [30]. In addition to self-reports, ChANGE-HD also extends beyond these studies by recording participant data from parents’ reports as well. Lastly, our neuroimaging protocols have been updated to conform to other large-scale studies of children and adolescents (see *Magnetic Resonance Imagine* section below) [31]. We note that although ChANGE-HD is distinct in many ways, we maintain several measures used in previous studies, such as the core neuroimaging scans to measure neural volume (including T1 and T2-weighted scans), verbal fluency tests, CAG repeat length assessments, and NfL quantification.

The goal of ChANGE-HD is to model development (versus degeneration) by focusing on individuals whose brain changes likely reflect neurodevelopment rather than disease progression. The broader objectives include:

1. **Using structural and functional MRIs to monitor brain development over time**. A specific focus is given to striatal circuitry, due to its centrality to HD and its prominence in earlier data.
2. **Evaluate the relationship between brain structure and cognitive, behavioral, and neurologic assessments**. Here, we aim to elucidate the precise effects of *mHTT* on cognition.
3. **Investigate the link between CAG repeat length and clinical (motor scores, cognitive evaluations etc.) and biological (neuroimaging, biomarkers) measures**. This includes evaluation of NfL as an appropriate marker of neurodegeneration in HD.
4. **Investigate the putative roles of genetic variants on development.** CAG repeat length is the most significant driver of symptom progression in individuals with *mHTT*. However, there still exists variation in the age of motor onset in individuals with similar CAG repeat lengths. This fact has motivated genome-wide association studies to identify variants that delay (e.g., *FAN1*) or accelerate (e.g., *MSH3*) the age of motor onset in patients with HD [32–34]. Several of the identified variants exist on genes implicated in DNA repair [33,35], ultimately exacerbating or preventing somatic instability. While these genes are typically studied in the context of HD neurodegeneration, several play a role in disorders associated with altered neurodevelopment, such as schizophrenia and autism [36], and HD and muscular dystrophy [35]. Using blood or saliva samples obtained at each visit, we will explore whether these genetic variants accelerate or delay development.

The study objectives will be achieved by improving upon the Kids-HD infrastructure, including adding more study sites and adding more precise measures (e.g., Q-Motor). We expect the study to validate pre-manifest biomarkers of neurodegeneration and their relationship with the age of motor symptom onset. We also expect to reveal novel and potentially opposite effects of these biomarkers on neurodevelopment:

- **Molecular/Genetic**
  ◦ Neurofilament light [18]
  ◦ Genetic modifiers, including *MSH3*, *FAN1*, *MLH1*, *PMS1*, *PMS2*, *MLH3*, *TCERG1*, *GPR161*, *RRM2B*, and *CCDC82* [33]
- **Neuroimaging**
  ◦ Striatal, cortical, and white matter volume [17,37,38]
  ◦ Cortical surface area, folding index, and curvature [17]
  ◦ White matter integrity in corpus callosum and corticostriatal pathways [39]
  ◦ Prefrontal-striatal, motor-striatal, and default mode network resting state functional connectivity [40]
  ◦ Hub organization [41]
  ◦ Network segregation [41]
- **Cognitive**
  ◦ General intelligence [38,42]
  ◦ Cognitive processing speed [42]
- **Motor**
  - Finger tapping variability (increased irregularity in rhythm) [37,43]
  - Grip force fluctuations [37,43]
  - Frequency of repetitive movements [37,43]

## Methods

### Ethics (protocol approvals, consents, blinding, and safety)

Study approval and centralized oversight were provided by the WIRB-Copernicus Group (WCG; formerly WIRB; study number: 1269202; IRB Tracking Number: 20192908). WCG ensures compliance with federal regulations, ethical guidelines, local laws, participant safety, and study integrity. The study was conducted according to the principles expressed in the Declaration of Helsinki. Consent (or assent) procedures vary based on the age range of our participants. Assent is a simplified version of the consent document presented in age-appropriate language:

- **Ages 6–7:** The assent form is verbally explained to minor participants, and they will choose whether to provide verbal assent. If verbal assent is provided, it is indicated on the physical consent document by the research team memeber who explained the assent form to the minor participant. A parent or legal guardian reviews and chooses whether to provide full written consent on the participant’s behalf.
- **Ages 8–11:** Literate participants have the assent form verbally explained to them, read, and decide whether to provide written assent. A parent or guardian then decides whether to provide written consent on their behalf.
- **Ages 12–17:** Both the participant and their parent or legal guardian review the full consent form with a research team member and decide whether to provide written consent.
- **Ages 18–30:** Adult participants review the full consent form with a research team member and decide whether to provide written consent on their own behalf.

Collecting predictive genetic data for HD in individuals below 18 years of age raises the risk of confidentiality and privacy breaches, the results of which can have highly negative psychological consequences on those individuals. Thus, a study of this kind warrants commensurate security measures. In accordance, all testing is performed in a double-blind manner and all research team members who interact with participants or their families remain blind to the participant’s genetic status. Those who have previously undergone genetic testing are asked not to disclose their results. Team members responsible for data analysis only have access to de-identified data and do not have any contact with participants or their families. At no point, including after the study’s completion, will any team member have access to both identifying information and genetic status. Genetic results are never shared with participants, their families, medical providers, or anyone with access to identifying information. Additionally, participants are free to withdraw from the study at any time. Data obtained prior to the time of withdrawal are retained, unless the participant or their caregiver specifically requests that all collected data be discarded. To verify that our protocols ascend to the appropriate level of security required to protect the records of all participants giving genetic samples in our study, the ChANGE‐HD study protocols were submitted to several HD patient advocacy groups, including Help4HD International, the Huntington’s Disease Society of America (HDSA), and the Huntington’s Disease Youth Organization (HDYO). All groups have reviewed and endorsed our security measures.

### Design

The ChANGE-HD study employs an accelerated longitudinal design (ALD), which is widely regarded as the gold standard for assessing developmental changes in brain development in children [44]. Instead of a traditional longitudinal design with two necessary time points separated by a specified interval, all visits across all participants are combined to create the largest possible pool of assessments. This complementation across hundreds of participants allows for data to be obtained across a wide range of YTO and allows more flexibility for individuals to participate as they can have their follow-up visits anytime nine months after their previous visit, if desired. This design allows us to combine cross-sectional and longitudinal approaches by recruiting participants across the desired age range. The clinical and imaging protocols are modeled after the ‘Adolescent Brain Cognitive Development’ (ABCD) study, providing a well-established framework for cognitive, behavioral, and MRI assessments in a longitudinal multi-site setting [31]. While the protocol has been designed such that several of the measures are standardized for comparison across the range of 6–30 years-of-age (e.g., NIH Toolbox instruments), other measures, such as the Quantitative-Motor (Q-Motor) require age-correction. For every visit, informed consent is obtained, screening procedures are performed, and, if the participant is of childbearing potential, a pregnancy test is performed. The following assessments are completed at each study visit (Table 2; see S2 Table for more details):

**Table 2:** Procedures performed at each ChANGE-HD visit.

	Baseline Visit 1	Visit 2	Visit 3	Visit 4
Screening	•	•	•	•
Consent	•	•	•	•
Demographics	•	Review and update if changed		
Vitals	•	•	•	•
Urine pregnancy screen *	•	•	•	•
Blood draw	•	•	•	•
Blood processing	•	•	•	•
Saliva **	•			
Birth history	•	Review and update if not collected previously		
Medical history	•	Review and update if changed		
Family history	•	Review and update if changed		
Medications	•	Review and update if changed		
Behavioral protocol (Self and Proxy)	•	•	•	•
Cognitive protocol	•	•	•	•
Motor protocol	•	•	•	•
MRI	•	•	•	•

* All females of child-bearing potential will be tested for pregnancy; ** Only completed at Visit 1 if blood draw is rejected; Details of all these measures are provided in S2 Table.

### Sites

The University of Iowa serves as the primary site for the ChANGE-HD study and received IRB approval from the WCG to begin recruitment on 11/18/2019. Four additional sites were added, including the Children’s Hospital of Philadelphia (CHOP), Columbia University, the University of Texas at Houston, and the University of California, Davis. Site were approved for recruitment by WCG on 11/16/2020, 11/16/2020, 5/12/2020, and 4/16/2020, respectively. Vanderbilt University was added to the study and received approval to recruit participants on 11/14/2024.

### Study period and recruitment goals

ChANGE-HD was originally planned as a 5-year study, aiming to complete 1,500 assessments of 400 participants by the end of the fifth year. However, the COVID-19 pandemic delayed study progress by causing significant disruptions in recruitment and scheduling across the five original study sites and required us to update our timelines after Year 2 (Table 3). As a result, the assessment period, including pre-COVID data collection, was extended to approximately seven years and our recruitment targets were changed to 450 participants across 1,030 assessments. Note that the extension allowed for the inclusion of new baseline visits, as well as additional repeat visits for those already enrolled. Actual recruitment values by site and year are shown in Table 4. Despite the disruptions of COVID-19, our most recent power analysis shows that we are still on track to achieve our study aims (see *Power analysis and sample size calculation*).

**Table 3:** Study timeline and recruitment targets. Numbers represent the targeted number of subjects and assessments, respectively.

	Year 1 ‘19‐20	Year 2 ’20‐21	Year 3 ’21‐22	Year 4 ’22‐23	Year 5 ’23‐24	Year 6 ’24‐25	Year 7 ’25‐26	Cumulative Total Target
**Target Total**	50/200	80/300	110/400	110/400	50/200			400/1500
**Revised Target after Year 1**		80/300	107/400	107/400	106/400			400/1500
**Revised Target after Year 2**		30/100	80/300	110/400	100/400	80/300		400/1500
**Revised Target after Year 3**		30/100	80/200	100/200	100/200	100/200	40/130	450/1030

**Table 4:** Recruitment by Site and Year. * - As of 02/20/2026.

	2020	2021	2022	2023	2024	2025	2026*	Total Recruitment
**The University of Iowa**	28	43	36	49	62	77	7	**302**
**CHOP**	3	10	27	40	44	55	8	**187**
**Columbia University**	7	16	26	19	27	24	0	**119**
**UT Houston**	0	13	36	38	30	36	0	**153**
**UC, Davis**	9	4	22	26	33	18	0	**112**
**Vanderbilt University**	0	0	0	0	63	63	5	**131**
**Cumulative Total**	**47**	**133**	**280**	**452**	**711**	**984**	**1004**	**1004**

### Participants

At baseline, approximately 450 total participants will be enrolled across all sites. Although annual visits are not required for an ALD design, retention is best with close follow-up, and subjects are asked to return annually. We aim to recruit an even distribution of subjects across age groups, with 225 participants in the range of 6–18 years-old and the remaining participants in the range of 19–30 years-old.

Inclusion criteria:

- Between 6–30 years old
- Fluent in English
- Have a biological parent or grandparent who has been genetically or clinically diagnosed with HD, indicating that the participant is at risk for HD (determined from participant or family report)
- Demonstrate an age-appropriate understanding of HD and its risks (If the parents indicate that their child is unaware, we will not enroll the participant until an age-appropriate discussion has occurred)

Exclusion criteria:

- A history of medical diagnoses that could potentially confound the study results (e.g., major head trauma, seizures, tumors, or any other major medical illness requiring ongoing care). If an aspect of their medical history obscures their symptoms as being directly attributable to HD (e.g., a concomitant congenital disorder) they are excluded from participation.
- Have Juvenile-onset HD (JOHD)
- MRI contraindications. Participants of childbearing potential must undergo a urine pregnancy test before their MRI. If positive, they are excluded from the MRI but may still complete other assessments.
- Manifestation of overt motor features of HD. This is ascertained by reporting from parents (or participants themselves if they are over 18) during a screening visit. If the participant or the parent reports that they are currently manifesting any motor symptom of HD, the participant is referred to a child or adult neurologist for clinical evaluation. These individuals are also invited to participate in the JOHD study if they were diagnosed with JOHD before the age of 21 (see below).

Participant recruitment is conducted across the United States. Potential participants along with their parents or grandparents are identified through local hospital or clinic records and HD registries. Recruitment platforms such as *Research Match*, *HD Trial Finder*, and *ClinicalTrials.gov* are used to share study information with the community. Additionally, study-dedicated social media channels and websites are maintained to disseminate updates. The study is also advertised through events, newsletters, social media, and websites of national HD organizations, including the HDSA, its state chapters, Help4HD International, National Youth Alliance (NYA) of HDSA, HDYO, and the Huntington Study Group (HSG).

### Power analysis and sample size calculation

Based on our findings from the Kids-HD sample, our central hypothesis is that GE participants will demonstrate altered trajectories in general brain size and cognition across YTO. Specifically, we predicted that GE individuals will demonstrate significantly larger brain volume (primary outcome measure) and stronger cognition scores (secondary outcome measures) prior to 20 YTO and significantly smaller brain volume and weaker cognition between 20−0 YTO. Using our updated recruitment target of 1,030 assessments across 450 participants, we estimated at least 90% power to reproduce our previous findings at p = .01 (except for cortical thickness; Table 5). Note that Table 5, which shows the results of our power analysis, includes functional and structural measures, but that our power analysis is primarily based on the structural findings from Kids-HD. Assuming 40% of participants are GE (as in the previous study), we can assume at least 400 visits from 180 unique genetically affected participants. The power analysis used the Kids-HD sample from Neema et al. (2024), and was based on 136 visits from 79 gene-expanded participants. Because the mean number of visits (and hence, statistical information) per participant will be higher in ChANGE-HD, a conservative estimate of the overall increase in statistical information (i.e., log-likelihood statistics) is obtained from the ratio of unique participants, which is 2.278. If the findings from Kids-HD hold, then the expected values of the analogous F statistics (Table 5) will increase by at least 2.278-fold. Standard power calculations using these projected F statistics yield results that exceed the above-claimed minimum statistical power.

**Table 5:** Tests used for hypotheses and power analysis (from Neema et al., 2024).

		Kids-HD				Change-HD		
**Measures**	**N**	**ndf**	**ddf**	**F**	**p**	**Projected F**	**Projected NCP**	**Power**
General Ability Index**	136	1	87.66	13.50	<0.001	30.78	29.445	0.997
Behavior composite**	135	2	120.27	10.91	<0.001	24.87	47.343	>0.999
PANESS**	128	1	46.12	8.92	0.005	20.34	18.933	0.956
Whole brain*	117	3	62.63	7.79	<0.001	17.76	49.491	>0.999
Cerebrum	117	3	62.55	7.80	<0.001	17.78	49.557	>0.999
Cortical gray matter*	117	1	99.90	12.22	<0.001	27.86	26.593	0.995
Cortical surface area	117	1	95.22	12.17	<0.001	27.75	26.468	0.994
Cortical thickness*	117	1	54.67	2.95	0.091	6.73	5.612	0.406
Cortical folding index	117	3	82.04	5.54	0.002	12.63	34.455	0.996
Cortical curvature index	117	1	62.27	10.49	0.002	23.92	22.559	0.983
Cerebral white matter*	117	3	51.46	9.84	<0.001	22.44	63.099	>0.999
Cerebellum	117	2	74.95	9.67	<0.001	22.05	41.540	>0.999
Striatum	117	5	102.37	13.98	<0.001	31.87	152.867	>0.999
Globus pallidus	117	5	80.15	16.22	<0.001	36.98	177.722	>0.999
Thalamus	117	1	102.87	7.47	0.007	17.03	15.871	0.916

Non-centrality parameter (NCP) is calculated from an F statistic by NCP = (F-(2F/ddf)-1)*ndf. These non-centrality parameters (NCP) refer to non-central chi-square distributions with the corresponding number of degrees of freedom. These are the large sample approximate distributions of the maximum likelihood test statistics under the minimum differences that cause rejection of the null hypothesis. These NCP values are a function of this minimum required difference and the contribution per participant to the analysis test statistic. ddf = denominator degrees-of-freedom, ndf = numerator degrees of freedom. * - primary outcome measures, ** - secondary outcome measures.

### Data collection and assessments

#### Biological samples.

Blood samples are collected via venipuncture at each visit using kits provided by Biospecimen Exchange for Neurological Disorders (BioSEND), an NIH-funded biorepository at Indiana University. If the blood draw is unsuccessful, a salivary sample is collected instead. After collection, samples are shipped to BioSEND for central storage and analysis. BioSEND will first perform genetic testing to determine the CAG repeat length within exon 1 of the *HTT* gene on chromosome 4. The number of repeats will be used to categorize study participants as either GE (≥ 36 repeats) or GNE (≤ 35 repeats). Additionally, plasma NfL concentrations will be measured using the *NF-Light® assay* with the *Simoa HD-1 analyzer* in collaboration with the Queen Square Institute of Neurology, University College of London, London, United Kingdom. Biosamples will become available to investigators via BioSEND after data collection, data analysis, and publication of primary outcome measures are complete in September of 2027.

#### Cognitive, behavioral, environmental, and motor measures.

As mentioned previously, the clinical assessment protocol is modeled after the ABCD Study [31], which tracked healthy children (9–10 years old) through adolescence. Although we are studying individuals who are both younger (6–9 years old) and older (25–30 years old), the ABCD study provides the gold standard model for large-scale studies of children and adolescents, which make up a large segment of our population of interest. Table 6 details the full assessment battery, including measures of cognition, behavioral health, environment & health, and motor function.

**Table 6:** ChANGE-HD assessments. MTTC = Maximum time to complete in minutes.

Domain	Test Name	Construct	MTTC	Digital	Validated Age Range/ Administered Ages
**Cognitive**	NIH Toolbox – Picture Vocabulary [45]	Language	4	Yes	• 3-85/ 6-30
	NIH Toolbox – Oral Reading Recognition [45]	Language	3	Yes	• 7-85/ 6 −30
	NIH Toolbox – Dimensional Change Task [45]	Executive function	4	Yes	• 3-85/ 6-30
	NIH Toolbox – Flanker Inhibitory Control [45]	Attention	3	Yes	• 3-85/ 6-30
	NIH Toolbox – List Sorting Working Memory [45]	Working memory	7	Yes	• 7-85/ 6-30
	NIH Toolbox – Picture Sequence Memory [45]	Episodic memory	7	Yes	• 3-85/ 6-30
	NIH Toolbox – Pattern Comparison Processing Test [45]	Processing speed	3	Yes	• 7-85/ 6-30
	NIH Toolbox – Rey Auditory Verbal Learning Task [45]	Verbal memory	3	Yes	• 8-85/ 6-30
	Controlled Oral Word Association [46]	Verbal fluency	5	No	• 6-69/ 6-30
**Motor**	Unified Huntington’s Disease Rating Scale (UHDRS) [47]	Motor	10-15	No	• 11 + / 6-30
	NIH Toolbox – 9-hole Pegboard Task [48]	Dexterity	4	Yes	• 3-85/ 6-30
	Quantitative-Motor [49]	Motor	45	Yes	• 6 + / 6-30
**Behavioral**	Achenbach System of Empirically Based Assessment [50]	Adaptive and maladaptive functioning	20	Yes	• Child Behavior Checklist Validated ages: 6–18 ◦ Administered ages: 6–17 ◦ Parent’s report • Youth Self-Report ◦ Validated ages: 11–18 ◦ Administered ages: 11–17 • Adult Behavior Checklist ◦ Validated ages: 18–59 ◦ Administered ages: 18–30 • Adult Self-Report ◦ Validated ages: 18–59 ◦ Administered ages: 18–30
	Urgency, Premeditation, Perseverance, Sensation Seeking, and Positive Urgency (UPPS-P) Behavior Scale [51,52]	Impulsivity	5	Yes	• Short version ◦ Validated ages: 14–72 ◦ Administered ages: 12–30 • Children’s version ◦ Validated ages: 7–13 ◦ Administered ages: 6–11
	Behavioral Inhibition/Behavioral Approach System (BIS/BAS) [53]	Inhibition/Reward seeking	5	Yes	• Ages 6–45/ 6–30
	Child and Youth Resiliency Measure, 5-pt scale versions [54]	Resilience	3	Yes	• Ages 6–17/ 6–17 ◦ proxy and self-report
	Adult Resiliency Measure, 5-pt scale versions [54]	Resilience	3	Yes	• 18 + / 18-30 ◦ proxy and self-report
	Sleep Disturbance Scale for Children [55]	Sleep	5-10	Yes	• 6 + / 6-30
	Sports and Activities Involvement Questionnaire [56]	Involvement in sports, music, hobbies	5-10	Yes	• 9-13/ 6-30
	Ohio State Traumatic Brain Injury Screen-Short [57]	Traumatic brain injury history	5-10	Yes	• 6 + / 6-30
	Substance Abuse and Substance Use Form (SASUF) [58]	Substance use/abuse	2	Yes	• 9 + / 12-30
**Environment and Health**	Neuro-QoL short forms [59]	Mental health	6-9	Yes	• Positive Affect and Well-being ◦ Validated Ages: 18+ ◦ Administered Ages 18–30 • Emotional and Behavioral Dyscontrol ◦ Validated Ages: 18+ ◦ Administered Ages 18–30 • Anger ◦ Validated ages: 8–17 ◦ Administered ages: 6–17
	Neuro-QoL (Quality of Life) short forms [59]	Social health	6-9	Yes	• Ability to Participate in Social Roles and Activities ◦ Validated Ages: 18+ ◦ Administered Ages 18–30 • Satisfaction with Social Roles and Activities ◦ Validated Ages: 18+ ◦ Administered Ages 18–30 • Social Relationship – Interaction with Peers ◦ Validated ages: 8–17 ◦ Administered ages: 6–17

In the Kids-HD study, cognitive evaluation relied heavily on the Wechsler scales. However, in alignment with new NIH guidelines seeking to improve the inter-rater reliability of multi-site cognitive outcomes, a protocol change was implemented to use the NIH Toolbox (Version 2) [45]. The NIH Toolbox is an iPad-based assessment tool designed to obtain comprehensive metrics from a single visit in the following domains: language, verbal fluency, executive function, attention, working memory, episodic memory, processing speed, and visuospatial intelligence. It offers several advantages, including being digital, standardized, and comprehensive, reducing variability in test results, improving the precision of cognitive measurements, and enabling comparisons across diverse ages and sites. The NIH Toolbox is also validated for use in individuals across the lifespan (ages 3–85; [45,48,59]) and is widely used to study neurological disorders and diseases, such as stroke [60] and Huntington’s disease [61,62], and other clinical populations [63]. Critically, the NIH Toolbox provides a domain-specific score based on all cognitive tests. These standardized cognitive domain scores will serve as our primary metric to assess cognitive function, although we will also explore the effects of gene expansion on each individual test.

Motor functions are assessed using the Unified Huntington’s Disease Rating Scale (UHDRS) motor battery, the NIH Toolbox Nine Hole Peg Test, and the Quantitative Motor (Q-Motor; primary outcome measure for motor performance) assessment. The Q-Motor battery assesses tapping speed and regularity of movements produced by index fingers, hands, and feet. It also quantifies grasping, lifting, and involuntary choreiform movements. The Q-Motor battery is also likely to be more sensitive to pre-manifest changes in motor performance than the UHDRS-TMS [64]. Altogether, it provides a comprehensive and precise evaluation of motor function that has been validated in children [30].

We measure behavioral metrics including participants’ adaptive and maladaptive functions, impulsivity, inhibition/reward seeking, and psychological resiliency using the Achenbach System of Empirically Based Assessment, the Urgency, Premeditation, Perseverance, Sensation Seeking, and Positive Urgency (UPPS-P) impulsive behavior scale, the Behavioral Inhibition/Approach System scale (BIS/BAS), and the Adult/Child-Youth Resilience measure, respectively. Finally, we also record participants’ physical, mental, and social well-being, sleep, the presence of sleep disorders, physical activities, history of traumatic brain injury, and substance use and abuse using Neuro-QoL (Quality of Life) [65], the Sleep Disturbance Scale for Children [55], the Sports and Activities Involvement Questionnaire [56], the Ohio State Traumatic Brain Injury Screen (short version) [57], and a substance abuse and use form (SASUF, made in-house) [58], respectively. All data collected under this protocol will be de-identified and uploaded to the NDA following data collection and analysis.

#### Magnetic resonance imaging.

Our main goal in designing the ChANGE-HD MRI protocol was to improve on the Kids-HD protocol, giving us the best chance of reproducing previous findings [17] and revealing new ones. To this end, the MRI protocol was designed to collect high resolution structural and functional MRI data within the time span of a typical research MR exam (~1 hour). To harmonize data across scanning sites, the scanning protocol was adapted from the ABCD study [31]. We used 3 Tesla research scanners to collect T1-, T2-, and diffusion-weighted, and resting-state functional MR brain images (see Table 7 for sequence parameter details). These sequences have been adapted and optimized for all six sites across Philips, General Electric, and Siemens MR scanner platforms.

**Table 7:** ChANGE-HD harmonized imaging scanning parameters for 3T scanners. FOV = field of view, TR = repetition time, TE = echo time, TI = inversion time, MP = MPRAGE, GR = spoiled gradient echo. VUMC = Vanderbilt University Medical Center.

Siemens (CHOP/ UC Davis)	Matrix	FOV	%FOV phase	Resolution (mm)	TR (ms)	TE (ms)	TI (ms)	Flip Angle (deg)	Parallel Imaging	Multiband Acceleration	Phase partial Fourier	b-values	Acquisition Time
**T1**	256x256x176	256x256	100	1.0x1.0x1.0	2500 (MP)	2.90	1060	8	2x	Off	Off	N/A	7:26/ 5:48
**T2**	256x256x176	256x256	100	1.0x1.0x1.0	3200	564/545	N/A	120/ Var	2x	Off	Off	N/A	4:52/ 4:42
**Diffusion**	140x140x81/ 122x122x78	240x240/ 241x241	100	1.7x1.7x1.7\ 2.0x2.0x2.0	4100/ 4170	88/ 103	N/A	88/ 90	Off	3	6/8	**CHOP**: 500 (6), 1000 (20), 2000 (20), 3000 (64). **UC Davis**: 500(6), 1000(15), 2000(60)	13:57/ 7:05
**fMRI**	90x90x60/ 86x86x60	216 x 216	100	2.4x2.4x2.4	800	30	N/A	52/67	Off	6	Off	N/A	5:00 x 4/ 4:08 x 4
Philips (**UT Houston**/ **VUMC**)	Matrix	**FOV**	**%FOV phase**	**Resolution (mm)**	**TR (ms)**	**TE (ms)**	**TI (ms)**	**Flip Angle (deg)**	**Parallel Imaging**	**Multiband Acceleration**	**Half Scan Factor**	**b-values**	**Acquisition Time**
**T1**	256x256x225	256x256/ 256x240	93.75	1.0x1.0x1.0	6 (GR)	3.0	1060	8	2 x	Off	Off	N/A	5:52
**T2**	256x256x256	256x256	100	1.0x1.0x1.0	2500	251.7/ 268	N/A	90	2.5 x	Off	Off	N/A	2:53/ 2:50
**Diffusion**	144x144x80	240x 240	100	1.7x1.7x1.7	6500	96	N/A	78	Off	3/ 4	0.603	500 (6) 1000 (15) 2000 (15) 3000 (60)	11:07/ 11:11
**fMRI**	96x96x60	230x230	100	2.25x2.25x2.4	800	30	N/A	52	Off	6	Off	N/A	5:00 x 4/ 5:15 x 4
General Electric (**Iowa**/ **Columbia**)	Matrix	**FOV**	**%FOV phase**	**Resolution (mm)**	**TR (ms)**	**TE (ms)**	**TI (ms)**	**Flip Angle (deg)**	**Parallel Imaging**	**Multiband Acceleration**	**Phase partial Fourier**	**b-values**	**Acquisition Time**
**T1**	256x256x208	256x256	100	1.0x1.0x1.0	2622 (MP)/ 2296 (MP)	2.96	1060	8	2 x	Off	Off	N/A	7:12/ 7:20
**T2**	256x256x208\ 256x256x204	256x256	100	1.0x1.0x1.0	3200/3202	60/ 55.7	N/A	Var/ 90	Off	Off	Off	N/A	6:28/ 4:58
**Diffusion**	140x140x81	240x240	100	1.7x1.7x1.7	9000/ 10000	102 \ 101.7	N/A	90	Off	2	5.5/8/ Off	**Iowa**: 500(6) 1000(15) 2000(15) 3000(60) **Columbia:** 3000 (96)	14:36/ 17:30
**fMRI**	96x96x60	307x307	100	2.4x2.4x2.4	800	30	N/A	52	Off	4/ Off	Off	N/A	5.00 x 4

As in the ABCD study, all images across sites were acquired in the axial plane because GE and Philips scanners may cause ghosting artifacts when acquired at oblique angles. Several improvements have been made to our MRI protocol since the Kids-HD study. While Kids-HD involved a single Siemens scanner at the University of Iowa, ChANGE-HD involves a mix of three vendors (Iowa: General Electric, CHOP: Siemens, Columbia: General Electric, UT Houston: Philips, UC Davis, Siemens, Vanderbilt: Philips). T1- and T2-weighted scan resolution has been increased by decreasing voxel sizes from 1.1 mm (T1) and 1.0x1.4x1.0 mm (T2) to 1.0 mm isotropic. To improve signal-to-noise ratio (SNR), we also increased the head coil channels from 12 in Kids-HD to between 32 and 48 (depending on scanner model) in ChANGE-HD.

Several improvements to resting-state functional scans were made. First, instead of acquiring one five-minute scan, we collected two scans (10 minutes total), each with opposite phase encoding directions. These improvements were made to reduce the effects of spurious noise and improve SNR [66], while reducing susceptibility effects [67]. Voxel and temporal resolution were also improved by decreasing voxel size from 3.4x3.4x4.0 mm to at least 2.4 mm isotropic and decreasing the TR from 2500 ms to 800 ms, respectively.

Finally, diffusion scanning was improved by decreasing voxel sizes from 2.0 to 1.7 mm isotropic and reducing distortion potential by lowering the echo time from 81 ms in Kids-HD to 69 ms in ChANGE-HD. Thus, the improvements in hardware and software represent a significant technological improvement between studies. Although scanning times differed by study site and participant visit, the approximate active scanning time was ~ 1 hour, including ~16 minutes for structural T1/T2 scans, ~ 18 minutes for diffusion scans, ~ 22 minutes for resting state scans, and ~2 minutes for BOLD field maps. Participants were given opportunities to take breaks and leave the bore, if needed.

During data collection periods, several quality control measures were followed to optimize scan quality. First, prior to study initiation, and once per year, each site was responsible for human phantom calibration. These calibrations were performed by designated credentialed imaging experts at each site (see S1 Table for each imaging expert). Second, each site’s imaging expert and team were required to regularly check scans for signs of poor scan quality, such as low tSNR or high resting state scan motion. In some cases, scans were repeated to ensure high image quality (e.g., the appearance of participant motion during resting state scanning). Third, deviations in protocol or changes to the study MRI system were noted by each site’s MR physicist. Post-data collection metrics of quality control will be reviewed to ensure that our final scans include only those with SNR of above 20, 45, 25, and 10 for anatomical, resting-state, b0, and b1000 diffusion scans, respectively. Resting state functional scans will be rejected if average framewise displacement exceeds 0.3 mm and frames will be censored if motion exceeds 0.3 mm.

All raw (uncensored, uncorrected, etc.) neuroimaging data collected under this protocol will be de-identified and uploaded to the NDA following data collection and analysis. Structural MRIs will be defaced to prevent participant identification.

#### Demographics and history.

Once cognitive, behavioral, motor, and environmental assessments have been administered, demographic and medical historical data are recorded. The parent or adult participant fills out demographic, family, and medical history forms in addition to completing a medication log. Finally, we record anthropometrics, including height, weight, head circumference, blood pressure, resting heart rate, and body temperature.

#### Data collection and management.

REDCap (*Research Electronic Data Capture*) is an encrypted, HIPAA-compliant web-based application used in ChANGE-HD for data entry and storage, eliminating the need for double data entry and verification. Internal audits of de-identified data are performed regularly to ensure accurate data entry and monitor interrater reliability.

### Data analysis

#### MRI processing and analysis.

Images from all sites are collected and imported into Flywheel, an infrastructure platform that allows researchers to manage, view, and analyze imaging data. Flywheel “gears” are containerized applications that automate reproducible analysis pipelines across all sites. Flywheel facilitates image sharing and automates analysis of volumetric, diffusion, and resting-state MR data. While standard quality control procedures are implemented during scanning, including image visualization, our pipelines are designed to provide quality control reports for each scan, allowing us to manually check and reject any scans identified as problematic (e.g., high motion, noise, etc.).

- **Structural/volumetric analysis:** We use *volBrain’s AssemblyNetZ* [68] and *FreeSurfer* [69] on anatomical T1-weighted volumes to obtain cortical volume, surface area, and thickness, and subcortical grey region volumes. Our primary volume measures include the basal nuclei and sensorimotor cortex. Of critical consideration for structural analysis is whether a single atlas should be used between younger and older participants. For example, the contrast between white and grey matter that is critical for *FreeSurfer* segmentation differs between children 5–10 years old and adults [70], introducing segmentation biases towards grey matter in children [71,72]. Additionally, head motion is greater in children, potentially, reducing the measured grey matter volume by 4–27% in this population [73]. Moreover, the reproducibility of FreeSurfer results in children in pediatric samples is lower than adults [74]. While these limitations may affect the longitudinal trajectory of our structural analyses, we note that the critical comparison is between GE and GNE individuals. Thus, it will be critical to identify group-specific differences in segmentation, such as potential differences in motion, rather than age-specific differences, to determine how gene overexpansion affects neurodevelopment.
- **Structural connectivity/Diffusion MR analysis**: We use diffusion MR and diffusion tensor imaging (DTI) to measure water molecule diffusion, allowing us to infer the integrity of white matter microstructure and the developmental maturity of brain tracts. High resolution, whole brain diffusion MR is acquired with multiple diffusion sensitivities (“b-values”). To correct geometric distortions in echo planar volumes, we acquire scans with opposing phase encoding directions (AP-PA) across all sites. Diffusion MR preprocessing is performed with the QSIPrep Flywheel gear [75]. QSIPrep is an automated pipeline that performs denoising, distortion correction, motion correction, and co-registration to T1 volumes and atlas spaces. Quality assurance (QA) reports on motion, noise, and image quality are generated at the subject level for review. The output of QSIPrep is then passed to two further diffusion MR analysis gears that measure diffusion parameters such as fractional anisotropy in regions of interest (ROIs) and tractography based structural connectome metrics.
- **Resting-state functional connectivity MRI analysis**: During resting state scans, participants are asked to remain awake and keep their eyes open while breathing and blinking normally and blood oxygen level dependent (BOLD) signal is measured. After signal is collected, the functional organization of the brain can be inferred by regressing the BOLD time series of multiple ROIs and network against each other, where positive correlations infer synchronous communication, negative values infer asynchronous communication, and near-zero or zero values infer little or no communication. Findings from resting state data are robust and reliable, revealing consistent functional networks [76] and strong intra-subject reliability [77]. We will use data from resting-state scans to analyze and understand functional neurodevelopment in GE subjects and how it compares to GNE subjects. Resting-state fMRI preprocessing is performed with fMRIPrep [78], which provides denoising, motion correction, susceptibility distortion correction, and alignment to each subject’s T1 anatomical image and atlas space. The preprocessed data are then analyzed using eXtensible Connectivity Pipeline Developmental Cognition and Neuroimaging (XCP-D) [79], which performs nuisance regression and temporal filtering and produces brain parcellated connectivity matrices. These matrices are generated using a cortical and subcortical atlas matched to the diffusion MR atlas, enabling direct comparison between structural and functional connectivity. For the ChANGE-HD study, the primary measures are pairwise functional connectivity values and network-level metrics related to striatal-cortical circuits.

#### Statistical analysis using YTO Models.

We will employ a YTO model, which provides a more detailed representation of disease-related changes (between 50 and 0 YTO) than an age-based analysis [15,16,18,23]. Our statistical analyses are derived from Langbehn et al. [80] and our own work [17] investigating neural and behavioral changes across YTO. This method is based primarily on data from individuals with CAG repeats between 41 and 56 [80] but extrapolation has generalized usefully to CAG repeats extending beyond this range (40, 57–70, etc.) [17]. Because individuals with between 36–39 CAG repeats represent an incomplete penetrance group, they will be excluded from the YTO analysis and will be examined separately in exploratory analyses. Individuals with >70 CAG repeats will also be excluded as they are likely to have already begun showing motor symptoms of HD and are likely classified as patients with JOHD. All hypotheses relate to either systematic group difference between GE and GNE individuals or to differences within the GE group driven by CAG expansion length and age (or years to onset as in the NfL analysis). Our analysis approach will include the following steps: 1) age and sex correction based on the data from healthy controls, 2), estimation of YTO, and 3) YTO modeling with testing for potential additional sex differences and sex by YTO interactions.

For each dependent variable, we apply statistical corrections to separate developmental effects of typical aging from those experienced by GE individuals as part of the disease trajectory. We perform this correction by modeling age and sex effects in GNE controls using a regression that includes within-subject and within-family random effects. The models incorporate both linear and potential non-linear age relationships. Nonlinearity is modeled flexibly via restricted cubic splines (“natural splines”). The degree of non-linearity (up to five degrees of freedom) is determined by choosing the model with the lowest Akaike Information Criterion (AIC), with tied AIC values (≤ 1.0) favoring the simpler model. We also test the model for sex and sex-by-age interactions, which are excluded from the final model if found to be nonsignificant. The fitted values for this model are then subtracted from the dependent value to complete the correction.

Estimation of age of motor symptom onset is calculated using the Langbehn model [80] shown below:


$$\begin{array}{c} Age\;of\;motor\;symptom\;onset=\text{2}\text{1}.\text{5}\text{4}-(\text{9}.\text{5}\text{5}\text{6}-\text{0}.\text{1}\text{4}\text{6}*CAG) \end{array}$$

YTO is then calculated by simply subtracting participants’ ages (in years) from age of motor symptom onset. (The abbreviation YTO, now established by precedent, stands for a slight misnomer. The value is the number of years from the mean age of onset for those with the given CAG length. It is not the individual’s number of years to onset—which is unknowable—and is slightly different from the individual’s expected years to onset, given that they have reached their current age without already experiencing onset.)

A limitation of this YTO approach is that it can only approximate the relationship between true years to HD onset and the outcome measures. As noted earlier, the YTO calculations are slightly biased estimates of participants’ expected years to onset. Furthermore, a person’s true years to onset has a probability distribution around the CAG-specific average. For concave relationships, such as those we typically expect in the neurodevelopment of brain volumes, substitution of an average value for a probability distribution causes at least slight underestimation of the predicted value that would be obtained from a model based on the true (but unknowable) number of years from onset (Jensen’s inequality).

We assess the relationship between YTO and age- and sex-adjusted outcome measures in GE individuals using an additional mixed effects regression, again including random effects for repeated measures of the same participant and for similarities between participants from the same family. Similar to the GNE model described above, the potentially nonlinear influence of YTO is modeled using restricted cubic splines with degrees-of-freedom chosen by AIC. Sex and sex interactions with YTO are tested and included in the model if significant. The mixed effect models are fitted by maximum likelihood and F-test degrees of freedom are estimated using the Satterthwaite approximation. The summary of the resulting model indicates a p-value for the effect of YTO, which, if significant, indicates a statistically meaningful difference of GE from GNE along the YTO trajectory. Finally, we plot these relationships and their 95% confidence intervals to determine where these differences occur along the trajectory (50−0 YTO)

To assess the relationship between age- and sex-adjusted neuroimaging variables (e.g., cortical grey and white matter volume, striatal volume, corticostriatal structural and functional connectivity, etc.) and clinical outcomes in GE individuals, we apply modeling techniques as described above. The models include random effects of subject and family, but exclude age and sex, as the values will have already been corrected using the data from GNE participants. Imaging measures are generally predictor variables, and clinical measures are the outcomes, reflecting the potential causal relationships between the two measurement classes. As in our previous work [17], we also split our GE participants into two groups with a cutoff of 20 YTO and test for differences in the imaging versus clinical relationships. This is based empirically on our work [17] showing divergence in neural and behavioral measures at this point in the YTO trajectory and because of work showing an upward trend in NfL at 20 YTO [18]. As described above, potential non-linear relationships between biomarkers and clinical measures are interrogated using restricted cubic splines (up to five degrees of freedom). For all analyses, a *p* value of < 0.05 is considered statistically significant.

In addition to our hypothesis-driven analyses, we also plan to perform detailed exploratory analyses to uncover novel neuroimaging biomarkers that both distinguish GE and GNE individuals and individuals with variations in HD-related single nucleotide polymorphisms (SNPs). For example, machine learning approaches, such as deep learning, have been applied to neuroimaging data from patients with neurodegenerative disorders to identify how those SNPs affect brain anatomy and function [81]. As mentioned above, SNPs in several genes explain variation in the eventual onset of motor symptoms in HD [32–34]. Using deep learning and other machine learning approaches as exploratory tools may reveal new mechanistic information about how these SNPs shape neurodevelopment and -degeneration.

### The ChANGE-Juvenile Onset HD (ChANGE-JOHD) Study

As part of the same study and funding mechanism, we are also conducting the “ChANGE-Juvenile Onset HD (ChANGE-JOHD) Study,” which will leverage the same infrastructure and multi-site design as the ChANGE-HD study. Although most HD patients typically manifest obvious motor signs between the ages of 40 and 50 (known as Adult-Onset HD, AOHD), a much smaller percentage of patients are diagnosed with motor features before the age of 21 (classified as JOHD) [27]. Patients with JOHD experience the same triad of cognitive, behavioral, and motor symptoms as those with AOHD, albeit with distinct phenotypes [82]. Specifically, JOHD patients tend to exhibit less hyperkinesia and more hypokinesia compared to AOHD. However, patients with JOHD experience CAG repeat expansion and symptom progression at a faster rate compared to patients with AOHD [83]. The progression of JOHD is theorized to involve several neuropathologic mechanisms, including reduced gestational neurogenesis and cell differentiation, neuronal migration, and synaptogenesis, as well as hyper and hypo-synaptic pruning during puberty [84]. However, due to its rarity, many aspects of the neurobiology and progression of JOHD are unclear.

While large-scale observational studies of AOHD have significantly advanced our understanding of HD, JOHD patients have been largely excluded from these efforts [85]. As such, large-scale longitudinal studies in JOHD are needed to deepen our understanding of the pathophysiology, as well as therapeutic strategies. When the Kids-HD study began at the University of Iowa in 2009, children at risk for HD who were already manifesting motor signs were followed as well. This was called the Kids-JOHD study (which ran in parallel to the Kids-HD study), whose primary objective was to deepen our understanding of the course and progression of JOHD. Cross-sectional analyses showed that patients with JOHD have significant subcortical degeneration shortly after diagnosis, along with reduced general measures of growth (such as weight and BMI) compared to GNE controls [21,86,87]. Furthermore, the longitudinal trajectory of disease severity is markedly hastened and amplified in JOHD compared to AOHD [88]. Despite these initial assessments from the Kids-JOHD study, larger-scale longitudinal studies in JOHD are still lacking. To this end, we have developed the first ever multi-site, prospective and comprehensive study of JOHD, leveraging the established infrastructure of the titular ChANGE-HD study to record clinical and neuroimaging metrics in this population. This study, which is considered separate from the ChANGE-HD but also follows an ALD, aims to investigate whether comprehensive longitudinal quantitative assessments of motor skills, cognition, and neuroimaging in JOHD patients can yield reliable biomarkers of disease progression. A total of 40 patients with JOHD will be recruited. In the first year, 20 patients with JOHD (3–4 per site) will complete baseline assessments, with the remaining 20 patients undergoing baseline assessments in Year 2. Year 2 will also include annual follow-up visits for the first 20 patients. In Year 3, follow-up visits will be conducted for the second group of 20 patients. As of the time of this writing, we have collected 49 assessments from 34 participants. For the study, the comparator group will include GNE participants recruited in the ChANGE-HD protocol. Data and biospecimens collected from the ChANGE-JOHD study will be shared separately from the ChANGE-HD study after data collection and analysis of primary outcome measures are complete, which we estimate will occur in September of 2027. To participate, JOHD participants must:

- Be diagnosed with JOHD by a trained neurologist prior to the age of 21, based on motor symptomology (the participant’s genetic status is confirmed after consent is obtained).
- Be between 6–30 years old at baseline. Thus, even though the diagnosis is made prior to 21, assessment for the study is not restricted to ages under 21.
- Not be bed-ridden, unable to communicate, or beyond travel conditions that are too strenuous for the individual.

## Discussion

ChANGE-HD is a longitudinal study designed to prospectively examine the neurodevelopmental phase of HD in premanifest at-risk children and young adults. The parallel ChANGE-JOHD study will also leverage the infrastructure of the ChANGE-HD study to investigate potential biomarkers of disease progression. Recruitment is currently underway at all six clinical sites. The data collected will provide crucial insights into the effects of *mHTT* and help uncouple neurodevelopmental changes from neurodegeneration.

Although recruitment is still ongoing, there are some prospects of how the results of ChANGE-HD may support the neurodevelopmental theory of HD. One such way is through antagonistic pleiotropy, as investigated by Neema et al. (2024) using the neuroimaging data of Kids-HD [17]. Therein, cortical hypertrophy and cognitive advantage were identified in the GE cohort very far from motor onset. If similar results are identified with the broader sample of ChANGE-HD, this would provide further support for the theory that *mHTT* confers developmental advantages antecedent to the degenerative phase.

Linked to this proposal is the theory of glutamatergic excitotoxicity in HD. It is a well-established fact that many neurological disorders are grounded in excessive neurotransmission through glutamatergic pathways which can then cause the development of epilepsy and/or cortical and subcortical volumetric atrophy [89–92]. Cortico-striatal pathways, which are highly implicated in the pathogenesis of HD, are primarily glutamatergic in nature [93]. If cortical hypertrophy is indeed characteristic of the developmental phase of HD, it is possible that a concomitant increase in glutamatergic neurotransmission is present as well. If this is true, there may be a direct causal link between developmental hypertrophy and downstream atrophy of striatal volume. Although neurochemical data, which we are not collecting, will be needed to confirm this hypothesis, the ChANGE-HD study is designed to test several assumptions of the glutamatergic toxicity model through our collection of high-quality longitudinal neuroimaging (structural and functional) data. For example, we will determine whether abnormalities in corticostriatal resting state functional connectivity and white matter integrity can be observed during the neurodevelopmental, as well as neurodegenerative, phases of the disease.

Like other HD studies, the ultimate goal of the ChANGE-HD study is to guide the design of interventions to prevent or mitigate HD symptoms. Reproducing the findings of early-life advantages in GE individuals observed in the Kids-HD sample [17] would bolster support for the antagonistic pleiotropy theory of HD and caution against mutant Huntington downregulation early in the disease process. Indeed, if the gene confers a benefit, then such treatments may come at a cost before neurodegeneration has begun. We also expect that studying the disease before brain function decline will reveal new neuroimaging patterns that predict neurodegeneration. For example, outlining the time course of motor and frontal resting state functional connectivity patterns may reveal when corticostriatal hypertransmission begins to negatively affect behavior. Pharmaceuticals to alter glutamatergic concentrations associated with these connectivity patterns may be a promising line for therapeutics. Finally, we will investigate how genetic modifiers (i.e., *FAN1*, *MLH1*, *PMS1*, etc.) affect the trajectory of the disease process before neurodegeneration begins. While these modifiers are related to the continued expansion of CAG repeats (somatic expansion) and well-studied later in the disease process, it is unclear how they contribute to neurodevelopment. Linking these modifiers to neurodevelopment may reveal important neuropathological insights or uncover new interventional targets.

## Conclusions

The ongoing ChANGE-HD study is the only multi-site longitudinal study in the world specifically designed to prospectively examine the neurodevelopmental effects of *mHTT* on children, adolescents, and young adults at risk for HD. While the study has broad applications, its primary objectives are to identify early neurodevelopmental changes, deepen our understanding of disease pathogenesis, and guide the design of therapeutic interventions. Together with the prospective ChANGE-JOHD study, ChANGE-HD will provide valuable insights into key aspects of disease progression in HD.

## Supporting information

S1 Table Full list of ChANGE-HD authors. (DOCX)

S2 Table Assessments performed as part of the ChANGE-HD Protocol. (DOCX)
