# Supplementary material for: Childhood to Adult Neurodevelopment in Gene-Expanded Huntington’s Disease (ChANGE-HD): A prospective longitudinal neurodevelopmental study of Huntington’s disease
Source: PLoS One. 2026 Jun 25;21(6):e0336088. doi: 10.1371/journal.pone.0336088 (PMC13298748; doi:10.1371/journal.pone.0336088)
Supplement: S1 Table — (DOCX) [file pone.0336088.s001.docx]

|  | | |
| --- | --- | --- |
| **Site** | **Team member** | **Title/role** |
| University  of Iowa | Peggy Nopoulos, MD | PD / PI |
|  | Amy L. Conrad, PhD | Clinical Expert / Co-Investigator |
|  | David Moser, PhD, ABPP | Clinical Expert / Co-Investigator |
|  | Douglas Langbehn, MD, PhD | Primary Biostatistician / Co-Investigator |
|  | Vince Magnotta, PhD | Imaging Expert / Co-Investigator |
|  | Michael Freedberg, PhD | Research Scientist / Co-Investigator |
|  | Mohit Neema, MBBS, MA | Research Scientist / Co-Investigator |
|  | Eric Axelson, BS | Image Processing Specialist |
|  | Sonia Slevinski, MS, NCC | Primary Research Coordinator |
|  | Keara Turkington | JOHD Site Coordinator |
|  | Lauri Jennisch | ChANGE Site Coordinator |
|  | Steve Slevinski | Data base manager |
| Children’s Hospital of Philadelphia | Timothy P.L. Roberts, PhD | Imaging Expert / Co-Investigator |
|  | Jeffrey I. Berman, PhD | Imaging Expert / Co-Investigator |
|  | Lisa Blaskey, PhD | Clinical Expert |
|  | Shana Ward | Site coordinator |
| Columbia University Medical Center | Ashwini Rao, EdD | Clinical Expert / Co-Investigator |
|  | Sachin Jambawlikar, PhD | Imaging Expert / Co-Investigator |
|  | Mia Parker | Site Coordinator |
| University of Texas health Science Center  at Houston | Erin Furr Stimming, MD | Clinical Expert / Co-Investigator |
|  | Nivedita Thakur, MD | Clinical Expert |
|  | Natalia P. Rocha, PhD | Clinical Expert |
|  | Khader Hasan, PhD | Imaging Expert / Co-Investigator |
|  | Brittany Duncan | Site Coordinator |
| University of California Davis Medical Center | Alexandra O’Neill Duffy, DO | Clinical Expert / Co-Investigator |
|  | Costin Tanase, PhD | Imaging Expert / Co-Investigator |
|  | Fernando Rodriguez | Site Coordinator |
|  | Isabella Knott | Site Coordinator |
| Vanderbilt University | Katherine McDonell, MD | Clinical Expert / Co-Investigator |
|  | Kelly Watson, PhD | Clinical Expert |
|  | Kilian Hett, PhD | Imaging Expert / Co-Investigator |
|  | Isabelle Taylor | Site Coordinator |
|  | Brandon Low | Site Coordinator |
| George Huntington Institute  *Muenster, Germany* | Ralf Reilmann, MD | Research Consultant, Q-Motor & Q-Cog |
|  | Robin Schubert, MSc | Research Consultant, Q-Motor & Q-Cog |
| University College London, UK | Edward Wild, MD, PhD | Research Consultant. NfL |
