# Supplementary material for: Childhood to Adult Neurodevelopment in Gene-Expanded Huntington’s Disease (ChANGE-HD): A prospective longitudinal neurodevelopmental study of Huntington’s disease
Source: PLoS One. 2026 Jun 25;21(6):e0336088. doi: 10.1371/journal.pone.0336088 (PMC13298748; doi:10.1371/journal.pone.0336088)
Supplement: S2 Table — (DOCX) [file pone.0336088.s002.docx]

|  | | |
| --- | --- | --- |
| Instrument Title in REDCap | Full Instrument Name | Focus of Instrument |
| Demographics | Demographics | Collects information about gender, age, handedness, and race |
| Vitals | Vitals | Collects information about height, weight, blood pressure, and head circumference |
| Birth History | Birth History | Collects information about participant’s birth, information about mother’s pregnancies, and participant’s early life milestones |
| Family History | Family History | Collects information about familial health and medical history |
| Med History | Medical History | Collects information about the participant’s medical history and diagnoses |
| Medications | Medications | Collects information about medications the participant is taking and changes in medications over the course of their participation |
| Osu Tbi Steps 1-3 | Ohio State University Traumatic Brain Injury screening | These collect information about any head or neck injuries or trauma that the participant may have experienced |
| School and Home | School and Home | Collects information about the participant’s level of education, grades, parents’ education, parents’ occupations, and family’s socioeconomic status |
| Sports and Activities Survey | Sports and Activities Survey | Collects information about sports, groups, and hobbies the participant engages in |
| Functional Capacity | Functional Capacity | Collects information about the participant’s ability to fulfil daily needs and routines, work, tend to household chores, care for themselves, complete basic tasks, and operate independently |
| Sdsc | Sleep Disturbance Scale for Children  *Administer to all ages despite “Children being in name”* | Collects information about the participant’s sleep habits, sleep disruptions, and quality of sleep |
| Upps P Child 6-11 Self | UPPS-P Impulsive Behavior Scale  *Only administer to children 6-11* | Collects information about risk-taking and impulsive behavior |
| Bis Bas Self | Behavioral Inhibition / Behavioral Approach System | Collects information about the participant’s motivation and inhibition |
| Sasuf 12 30 Self | Substance Abuse and Substance use Form  *Self-report, only administer to participants 12-30* | Collects information about the participant’s use of substances. This assessment is only given to participants who are aged 12 and up. |
| Upps P 12 30 Self | UPPS-P Impulsive Behavior Scale  *Only administer to participants 12-30* | Collects information about risk-taking and impulsive behavior |
| ARM R_18_30_self | Adult Resiliency Measure  *Self-report, only administer to participants 18+* | Collects information about the participants feelings about and interactions with others from the participant’s perspective |
| PMK ARM R_18_30_proxy | Adult Resiliency Measure  *Proxy report, only administer to participants 18+* | Collects information about the participants feelings about and interactions with others from someone else’s perspective |
| Youth_CYRM_10_17_self | Youth Resiliency Measure  *Self-report, only administer to children 10-17* | Collects information about the participants feelings about and interactions with others from the participant’s perspective |
| Youth_PMK-CYRM_10_17_proxy | Youth Resiliency Measure  *Proxy report, only administer to children 10-17* | Collects information about the participants feelings about and interactions with others from someone else’s perspective |
| Child_CYRM_6_9_self | Child Resiliency Measure  *Self-Report, only administered to children 6-9* | Collects information about the participants feelings about and interactions with others from the participant’s perspective |
| Child_PMK_CYRM_6_9_proxy | Child Resiliency Measure  *Proxy report, only administer to children 6-9* | Collects information about the participants feelings about and interactions with others from someone else’s perspective |
